# High-Risk Human Papillomavirus Clearance with a *Coriolus versicolor*–Based Vaginal Gel

**DOI:** 10.3390/diseases14050156

**Published:** 2026-04-29

**Authors:** Haticegul Tuncer, Sevinj Hajiyeva, Betul Gungor Serin, Muhammed Onur Atakul, Ali Can Gunes, Taylan Onat, Utku Akgor, Derman Basaran, Zafer Selcuk Tuncer, Murat Gultekin

**Affiliations:** 1 Department of Obstetrics and Gynecology, Hacettepe University, Ankara 06050, Turkey; drhaticegultuncer@gmail.com (H.T.); betulgungorserin@gmail.com (B.G.S.); onuratakul@hacettepe.edu.tr (M.O.A.); dr.acgunes@gmail.com (A.C.G.); 2 Faculty of Medicine, Hacettepe University, Ankara 06050, Turkey; sevinchaciyeva620@gmail.com; 3 Sincan Training and Research Hospital, Ankara 06934, Turkey; taylan.onat@sbu.edu.tr; 4 Division of Gynecologic Oncology, Department of Obstetrics and Gynecology, Hacettepe University, Ankara 06050, Turkey; utkuakgor@gmail.com (U.A.); dermanbasaran@gmail.com (D.B.); zstuncer@hacettepe.edu.tr (Z.S.T.)

**Keywords:** human papillomavirus, cervical cancer, HPV clearance, cervical cytology, *Coriolus versicolor*, vaginal gel, non-ablative therapy

## Abstract

**Background/Objectives**: Non-ablative local therapies are increasingly used in the conservative management of human papillomavirus (HPV) infection. *Coriolus versicolor*, an immunomodulatory medicinal mushroom, is one such approach. This study aimed to investigate the effect of a *Coriolus versicolor*–based vaginal gel on HPV clearance and cervical cytological outcomes. **Methods**: This retrospective cohort study included 600 women with cervical HPV infection (300 treated with a *Coriolus versicolor*–based vaginal gel and 300 receiving standard follow-up). Baseline and six-month follow-up assessments included HPV DNA testing and cervical cytology. **Results**: Baseline demographic characteristics, HPV genotype distribution, infection type, and cytological findings were comparable between the groups. Overall HPV clearance was significantly higher in the treatment group than in the controls (89.3% vs. 44.7%, *p* < 0.001). Complete clearance of high-risk HPV genotypes, including HPV 16 (77.0% vs. 25.4%, *p* < 0.001) and HPV 18 (73.9% vs. 18.5%, *p* = 0.017), was also significantly more frequent among treated women. Cytological normalization occurred more often in the treatment group (88.4% vs. 60.4%, *p* < 0.001). Multivariable analysis identified use of the vaginal gel as the strongest independent factor associated with HPV clearance (adjusted odds ratio [aOR] = 10.19; 95% confidence interval [CI]: 3.52–29.47; *p* < 0.001). **Conclusions**: Treatment with a *Coriolus versicolor*–based vaginal gel was associated with significantly higher rates of high-risk HPV clearance and cervical cytological normalization. These findings suggest that this therapy may represent an effective adjunct in the conservative management of HPV infection; however, randomized controlled trials are warranted to confirm these results.

## 1. Introduction

Human papillomavirus (HPV) is the most prevalent sexually transmitted infection worldwide, with the majority of sexually active individuals acquiring the virus at some point during their lifetime. Over 200 HPV genotypes have been identified, with 14 classified as high-risk types that are strongly associated with nearly all cases of cervical intraepithelial neoplasia and cervical cancer in women [1]. Most HPV infections and related low-grade cervical abnormalities resolve spontaneously without medical intervention [2]. However, some infections persist. Factors contributing to persistence include specific viral genotypes, elevated viral load, age at initial diagnosis, vaginal microbiome imbalances (dysbiosis), and immune suppression. Persistent infection with high-risk HPV types substantially increases the risk of progression from early lesions to high-grade precancerous changes and invasive cervical cancer [3,4].

Cervical cancer remains a major cause of illness and death worldwide. Persistent infection with “high-risk” HPV types is the main cause [5]. As of 2022, cervical cancer ranked as the fourth most prevalent cancer among women globally in terms of both incidence and mortality, accounting for approximately 660,000 newly diagnosed cases and 350,000 deaths worldwide [6]. Cervical infection with high-risk genotypes, especially HPV-16 and HPV-18, leads to progression from transient infection to neoplasia and finally invasive carcinoma [5,7]. Recent global studies estimate HPV prevalence at about 11.7% for any type and 6.5% for high-risk HPV among women. These numbers show a substantial risk for disease progression [7]. Additional analyses confirm that persistent high-risk HPV infection is the main cause of cervical cancer in different regions [8].

Primary prevention through vaccination and screening has improved cervical cancer control. Still, gaps in coverage and adherence leave many women at risk [9,10]. Current guidelines promote HPV testing in “screen–triage–treat” protocols and recommend follow-up for HPV-positive women. Most algorithms focus on monitoring low-grade abnormalities. They reserve definitive therapy for confirmed higher-grade disease [9,10,11]. Studies show that most HPV infections clear within 12 to 24 months. Some persist, especially in adults or in the presence of cofactors. This has increased interest in adjunctive strategies to speed up viral clearance and repair mucosa without affecting safety [3,12,13].

Recently, non-ablative, locally applied products have been studied. These products aim to support cervical healing, maintain a healthy vaginal environment, and modulate local immunity. They may complement standard clinical follow-up in women with high-risk HPV infection and low-grade cervical abnormalities [14]. One approach uses *Coriolus versicolor* (syn. Trametes versicolor), a medicinal mushroom. Its protein-bound polysaccharides (PSK/PSP) help to enhance local immunity by affecting both innate and adaptive responses. These include the local activation of dendritic cells and macrophages, as well as cytokines involved in antiviral and antitumor actions [15,16,17]. This provides a basis for testing *Coriolus versicolor*–based vaginal gels as local aids to improve clearance of HPV and restore epithelial integrity.

Early clinical evidence is emerging. Papilocare^®^ vaginal gel (Procare Health Iberia SL, Barcelona, Spain) is a commercially available *Coriolus versicolor*–based vaginal gel with demonstrated efficacy in the normalization of HPV-related low-grade cervical lesions. The gel formulation includes hyaluronic acid, Asian centella, aloe vera, and alpha-glucan oligosaccharide, which possess tissue-regenerative and vaginal microbiota-protective properties. Additionally, *Coriolus versicolor*, Azadirachta indica, and carboxymethyl-β-glucan, present in Papilocare, are established ingredients that have demonstrated efficacy in clearing HPV-related cervical lesions. Papilocare vaginal gel primarily helps prevent virus-induced cervical lesions. It does this by promoting the re-epithelialization of the cervical transformation zone. Additionally, the gel supports normalization of virus-associated intraepithelial abnormalities, alleviates vaginal dryness, restores the integrity of the cervical-vaginal mucosa, reestablishes balanced vaginal microbiota, and enhances overall vaginal health. The PALOMA trial, a prospective, randomized, multicenter, open-label study with an observation-only control arm, was the first randomized clinical trial to demonstrate the efficacy of this vaginal gel in the normalization of HPV-related low-grade cervical lesions. It also showed higher HPV clearance rates versus control over six months [18]. Observational data from real-world use, such as PAPILOBS, indicate this multi-ingredient gel can achieve lesion regression and meaningful HPV clearance. These findings hold in routine practice, even in older women who often do not clear HPV spontaneously [19]. Sub-analyses in women aged ≥40 years show similar benefits and good safety, supporting validity across ages [20]. These studies do have limitations, including open-label designs and potential assessment bias. While the primary endpoints are generally consistent across studies—typically including lesion regression and HPV clearance at around 6 months—there is some heterogeneity in endpoint definitions, timing of assessments, and criteria used for HPV clearance. Still, their results support further rigorous evaluation in real-world groups relevant to clinical decisions [18,19,20].

This retrospective cohort study looks at women with cervical HPV infection in routine care. It measures the effectiveness of the *Coriolus versicolor*–based vaginal gel when added to standard follow-up. Specifically, it checks HPV clearance, cytologic and colposcopic results, and safety across subgroups. Findings are discussed in the context of current screening advice and new literature on non-ablative, immune-modulatory cervical therapies.

## 2. Materials and Methods

### 2.1. Study Design and Participants

This study was designed as a retrospective cohort study based on previously recorded clinical data. The study period extended from January 2023 to August 2025, and the research was conducted in the Department of Obstetrics and Gynecology at Hacettepe University Hospital, a tertiary referral center. Demographic and clinical data were retrieved from institutional medical records, including follow-up documentation, hospital archives, procedural databases, and comprehensive patient files containing data from both internal and external healthcare facilities. When necessary, missing or unclear retrospective information was supplemented through direct patient contact (via telephone).

A total of 600 patients were included in the study and divided into two groups. The Papilocare group consisted of 300 patients who received a *Coriolus versicolor*–based vaginal gel, while the control group included 300 patients who did not receive any systemic or topical pharmacological treatment and were managed with standard clinical follow-up without pharmacological intervention.

All participants underwent HPV DNA testing and cervical cytologic examination at baseline and at six months. Cervical cytology samples were collected using a Cervix-Brush^®^ cervical sampling device (CERVIX-BRUM, MDSS GmbH Schiffgraben 41,30175 Hannover, Germany), which was inserted into the cervical canal and rotated 360 degrees to obtain epithelial cells from both the ectocervix and endocervix. Immediately after sampling, the brush head was rinsed into a vial containing liquid-based cytology preservative solution (CY-PREP^®^ Pap Test Dual Filtration Cytology Preparation system, CY-REP Pap Test, GmbH Schiffgraben 41, 30175 Hannover, Germany). Cytological evaluation was performed according to standard laboratory protocols, and results were reported using the Bethesda System [21].

For HPV testing, cervical samples were collected using a sterile Copan eSwab^®^ (Copan eSwab, Copan Italia SpA, via Parotti 10, 25125 Brescia, Italy) collection and transport system. Following collection, samples were placed in transport medium and maintained at room temperature until processing. Specimens were subsequently transferred into PreservCyt solution (Hologic, Marlborough, MA, USA) and stored under appropriate conditions until analysis. DNA extraction was performed using the eMAG automated system (bioMérieux, Marcy l’Etoile, France), in accordance with the manufacturer’s instructions. HPV detection and genotyping were carried out using the Anyplex™ II HPV28 real-time PCR assay (Seegene, Seoul, Republic of Korea).

For analytical purposes, HPV genotypes were classified into five categories: HPV 16, HPV 18, other high-risk types (HPV 31, 33, 35, 39, 45, 51, 52, 56, 58, 59, 68, 73, and 82), intermediate-risk types (HPV 26, 53, and 66), and low-risk types (HPV 6, 11, 40, 42, 43, 44, 54, 61, 70, 72, and 81). In addition, HPV positivity was further categorized according to infection type as single-type HPV (presence of a single HPV genotype) and multiple-type HPV (presence of two or more HPV genotypes in the same sample).

Participants in the Papilocare group were instructed to apply the *Coriolus versicolor*–based vaginal gel according to the manufacturer’s recommendations. The treatment regimen consisted of one vaginal cannula administered daily for 21 consecutive days during the first month, followed by application on alternate days in the subsequent months. A high-dose regimen was defined as daily administration during the first three months of treatment. Some patients received this high-dose regimen for three consecutive months (corresponding to the use of three boxes), whereas others continued the same regimen for a total of six months (corresponding to the use of six boxes), thereby completing an overall treatment duration of six months. All patients receiving Papilocare were analyzed within the same treatment group regardless of treatment duration. Participants in the control group did not receive any topical or systemic treatment during follow-up.

All participants were advised to use condoms during sexual intercourse and to avoid vaginal douching or the use of vaginal deodorants throughout the study period. Compliance with these recommendations was assessed based on information recorded in routine follow-up visits. Based on retrospective follow-up records, only patients who adhered to these recommendations were included in the Papilocare group.

Diagnostic and therapeutic interventions, including colposcopy, targeted cervical biopsy, and excisional procedures such as loop electrosurgical excision procedure (LEEP), were performed based on clinical indications and in accordance with established cervical cancer screening and management guidelines [22].

Overall HPV clearance was defined as either complete or partial clearance. Complete clearance was defined as a negative HPV DNA test result or the absence of all baseline HPV genotypes. Partial clearance was defined as the disappearance of at least one baseline HPV genotype.

Cytological (smear) clearance was defined using analogous criteria. Overall clearance included both complete and partial responses. Complete clearance referred to normalization of cervical cytology, while partial clearance indicated regression to a lower-grade lesion.

Demographic, clinical, and behavioral data collected included participants’ age, educational level, Papilocare use and number of treatment boxes, pregnancy and parity history, menstrual regularity, oral contraceptive use, presence of chronic diseases, smoking status, HPV vaccination status, immunosuppression status, age at menarche, age at first sexual intercourse, baseline HPV genotype, and history of loop electrosurgical excision procedures.

Inclusion criteria comprised patients with a positive HPV DNA test and cervical cytology findings of normal cytology, atypical squamous cells of undetermined significance (ASC-US), atypical squamous cells—cannot exclude high-grade squamous intraepithelial lesion (ASC-H), low-grade squamous intraepithelial lesion (LSIL), high-grade squamous intraepithelial lesion (HSIL), or atypical glandular cells (AGC).

Exclusion criteria included the presence of sexually transmitted or symptomatic vulvovaginal infections, immunodeficiency or autoimmune disorders, current immunosuppressive therapy, pregnancy, prior total hysterectomy, or a history of gynecologic malignancy.

### 2.2. Ethics

The study was conducted in accordance with the principles of the Declaration of Helsinki and approved by the relevant ethics committees. The initial approval was obtained from the Hacettepe University Ethics Committee on 1 November 2022 (Project No: GO 22/1105; Decision No: 2022/18-08). The study was subsequently approved by the Ministry of Health Local Ethics Committee (Sincan Training and Research Hospital) on 23 December 2025 (Approval No: SEAH-BAEK 2025-151). Patient data were obtained from Hacettepe University Hospital, where written informed consent for the use of clinical data for scientific research purposes is routinely obtained from both outpatients and inpatients at the time of clinical evaluation.

### 2.3. Statistical Analysis

Statistical analyses were conducted using SPSS (Statistical Package for Social Sciences) (software version 25.0; IBM Corp., Armonk, NY, USA). Descriptive statistics were applied to summarize the demographic, clinical, virological, and cytological characteristics of the study population. Continuous variables were reported as mean ± standard deviation or median (interquartile range), as appropriate, whereas categorical variables were expressed as frequencies and percentages.

Comparisons between the Papilocare and control groups for continuous variables were conducted using the Mann–Whitney U test, as data did not consistently meet normal distribution assumptions. Categorical variables, including HPV genotype distribution, cytological categories, and clearance outcomes, were compared using the chi-square test. Fisher’s exact test was applied when expected cell counts were less than five.

HPV and cytological clearance outcomes were analyzed as complete, partial, and no clearance, as well as overall clearance (defined as the combination of complete and partial clearance). Group differences in clearance rates were assessed using chi-square tests.

To identify factors independently associated with overall HPV clearance, univariate and multivariable binary logistic regression analyses were performed. Variables included in the regression models were selected based on clinical relevance and prior evidence and comprised use of the *Coriolus versicolor*–based vaginal gel (yes/no), number of Papilocare treatment boxes (<3, ≥3), age (<30, 30–45, >45 years), pregnancy history (yes/no), menstrual status (regular, irregular, menopausal), oral contraceptive use (yes/no), presence of chronic diseases (yes/no), smoking status (current smoker/non-smoker), HPV vaccination status (vaccinated/unvaccinated), immunosuppression status (yes/no), age at menarche (≤15, >15 years), age at first sexual intercourse (≤18, >18 years), baseline HPV type (HPV 16/18/high-risk vs. other types), history of loop electrosurgical excision procedure (yes/no), and educational level (below university vs. university and above). Results were reported as odds ratios (ORs) and adjusted odds ratios (aORs) with corresponding 95% confidence intervals (CIs).

All statistical tests were two-sided, and a *p* value of <0.05 was considered statistically significant.

## 3. Results

### 3.1. Study Population and Baseline Characteristics

A total of 600 women with HPV infection were included in this retrospective analysis, comprising 300 women in the Papilocare group and 300 women in the control group. The mean age was 37 ± 9 years in the Papilocare group and 38 ± 9 years in the control group, with no statistically significant difference between groups (*p* = 0.279). Baseline demographic characteristics were comparable, indicating adequate group homogeneity (Table 1).

The distribution of HPV genotypes at baseline did not differ significantly between the two groups (*p* = 0.124). HPV 16 was the most prevalent genotype, detected in 37.7% of women in the Papilocare group and 39.3% of controls. HPV 18 positivity was observed in 7.7% and 9.0% of participants, respectively. Other high-risk HPV types accounted for 37.3% of infections in the Papilocare group and 33.3% in the control group. Intermediate-risk and low-risk HPV types were similarly distributed between groups (Table 1).

When HPV positivity was evaluated according to infection type, single-type HPV infection was identified in 51.0% of women in the Papilocare group and 47.7% in the control group, while multiple-type HPV infection was observed in 49.0% and 52.3% of participants, respectively, with no statistically significant difference between groups (*p* = 0.414) (Table 1).

Baseline cytological evaluation was available for 260 women in the Papilocare group and 271 in the control group. Normal cytology was present in approximately half of the participants in both groups (48.1% vs. 51.7%). The frequencies of ASC-US, ASC-H, LSIL, and HSIL were comparable, with no statistically significant differences observed at baseline (*p* = 0.171) (Table 1).

### 3.2. HPV Clearance Outcomes

Clearance analyses demonstrated significantly higher rates of HPV elimination in the Papilocare group compared with controls. For HPV 16, complete clearance was achieved in 77.0% of treated women, with an additional 14.2% showing partial clearance, resulting in an overall clearance rate of 91.2%. In contrast, the control group demonstrated complete and partial clearance rates of 25.4% and 16.1%, respectively, corresponding to an overall clearance rate of 41.5% (*p* < 0.001) (Table 2).

Similarly, for HPV 18, complete clearance was achieved in 73.9% of treated women, with an additional 17.4% showing partial clearance, resulting in an overall clearance rate of 91.3%. In contrast, the control group demonstrated complete and partial clearance rates of 18.5% and 25.9%, respectively, corresponding to an overall clearance rate of 44.4% (*p* = 0.001). Among other high-risk HPV types, complete clearance was observed in 76.8% of treated women, with an additional 12.5% demonstrating partial clearance, yielding an overall clearance rate of 89.3%; in the control group, the corresponding overall clearance rate was 46.0% (*p* < 0.001). Comparable trends favoring Papilocare were observed for intermediate-risk (88.2% vs. 41.7%, *p* = 0.012) and low-risk HPV types (82.9% vs. 48.4%, *p* = 0.018) (Table 2).

When stratified by HPV infection type, significant differences in clearance rates were also observed. Among women with single-type HPV infection, overall clearance was achieved in 83.7% of those treated with Papilocare compared with 35.0% in the control group (*p* < 0.001). In women with multiple-type HPV infection, overall clearance rates were even higher in the Papilocare group (95.2%) compared with controls (52.2%) (*p* < 0.001) (Table 2).

Overall HPV clearance, combining complete and partial responses, was achieved in 89.3% of women in the Papilocare group, compared with 44.0% in the control group, representing a statistically significant difference (*p* < 0.001) (Table 2).

### 3.3. Cytological Clearance Outcomes

Among women with abnormal baseline cytology, Papilocare treatment was associated with significantly higher rates of cytological normalization. For ASC-US lesions, overall clearance was observed in 87.8% of treated women, compared with 55.6% in the control group (*p* = 0.001) (Table 2). ASC-H lesions demonstrated overall clearance rates of 88.3% in the Papilocare group and 60.0% among controls (*p* = 0.042) (Table 2).

For LSIL, overall clearance occurred in 90.3% of women treated with Papilocare versus 65.0% in the control group (*p* = 0.028) (Table 2). HSIL clearance rates were also higher in the Papilocare group (89.5%) compared with controls (59.1%) (*p* = 0.006) (Table 2).

Overall smear clearance, combining complete and partial responses, was achieved in 89.9% of women in the Papilocare group compared with 59.5% in the control group, with the difference reaching statistical significance (*p* < 0.001) (Table 2).

HPV DNA detection and smear analyses revealed a statistically significant difference in clearance rates between the Papilocare and control groups (*p* < 0.001). This significant difference persisted across both complete and partial clearance conditions, as well as in the overall analysis (Table 2).

Of the patients with a diagnosis of HSIL, LSIL, or ASC-H who underwent the loop electrosurgical excision procedure, 73 belonged to the Papilocare group and 46 to the control group. In the Papilocare group, LEEP was performed in 12 of 24 patients (50.0%) with ASC-H, 25 of 41 patients (61.0%) with LSIL, and 36 of 54 patients (66.7%) with HSIL. In the control group, LEEP was applied in 12 of 24 patients (50.0%) with ASC-H, 16 of 41 patients (39.0%) with LSIL, and 18 of 54 patients (33.3%) with HSIL. Histopathological examination of LEEP specimens revealed cervicitis in 12 patients (63.2%) in the Papilocare group and 7 patients (36.8%) in the control group. CIN 1 was identified in 19 Papilocare-treated patients (65.5%) and 10 control patients (34.5%), CIN 2 in 15 (57.7%) and 11 (42.3%), and CIN 3 in 27 (60.0%) and 18 (40.0%) patients, respectively.

### 3.4. Multivariable Analysis

Multivariate logistic regression analysis identified use of the *Coriolus versicolor*–based vaginal gel as the strongest independent factor associated with overall HPV clearance in the combined cohort (adjusted OR 10.19; 95% CI: 3.52–29.47; *p* < 0.001). Other demographic and clinical variables, including age, smoking status, HPV vaccination, baseline HPV risk group, and cytological category, were not independently associated with clearance after adjustment (Table 3).

HPV infection type was associated with clearance in univariate analysis, with multiple-type HPV infections showing higher crude clearance rates compared with single-type infections (OR 1.75; 95% CI: 1.24–2.46; *p* = 0.002). However, this association did not remain statistically significant after adjustment in the multivariable model (aOR 0.50; 95% CI: 0.23–1.08; *p* = 0.078).

Among women treated with Papilocare, multivariable analysis demonstrated that none of the evaluated demographic, clinical, or behavioral parameters were independently associated with overall HPV clearance. No statistically significant associations were observed for age, pregnancy history, menstrual status, age at menarche, age at first sexual intercourse, HPV vaccination status, HPV infection type or baseline HPV risk group. All participants in the Papilocare group received a high-dose regimen, consisting of either three or six boxes of treatment, and no dose-related differences in HPV clearance were identified.

## 4. Discussion

**Figure 1:**
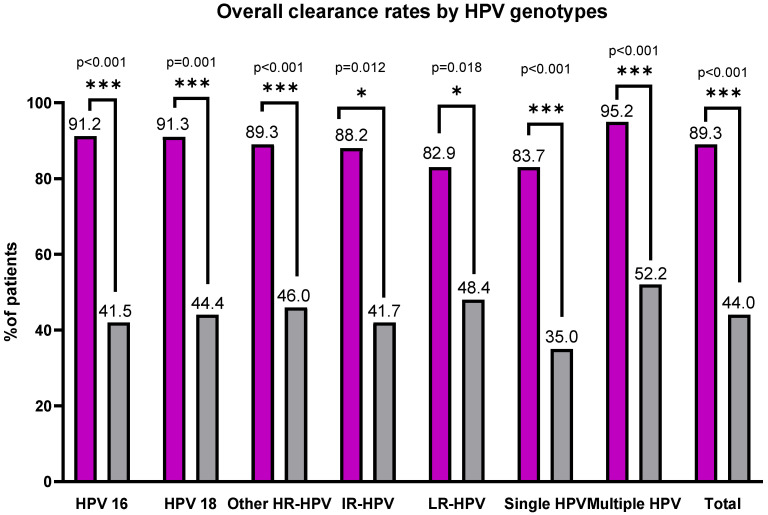
Overall (partial and complete) HPV clearance rates stratified by genotype and infection type in Papilocare versus control groups. HR: High-risk; IR: Intermediate-risk; LR: Low-risk. *** *p* ≤ 0.001; * *p* < 0.05 (0.001 < *p* < 0.05).

This retrospective study evaluated the effectiveness of a *Coriolus versicolor*–based vaginal gel in women with HPV infection and demonstrated significantly higher rates of both HPV clearance and cytological improvement in the Papilocare group compared with the control group. The beneficial effect was consistent across all evaluated HPV genotype categories, HPV infection types, and abnormal cytological subgroups. In particular, overall clearance rates for HPV 16 and HPV 18 reached 91.2% and 91.3%, respectively, in the Papilocare group, compared with 41.5% and 44.4% in the control group, respectively (Figure 1). Similar differences were observed for other high-risk, intermediate-risk, and low-risk HPV types, as well as for total HPV clearance (89.3% vs. 44.0%, *p* < 0.001) (Figure 1).

These observations suggest that the *Coriolus versicolor*-based gel may enhance regression of HPV-related cervical abnormalities, potentially by enhancing the local immunological environment [19,20]. *Coriolus versicolor* contains beta-glucans and polysaccharopeptides known to stimulate innate and adaptive immune responses, including macrophage activation, cytokine production, and enhancement of natural killer cell activity [16,23]. Such mechanisms offer a plausible explanation, in addition to the known mechanical barrier main effect and microbiome restoration, for the accelerated viral clearance observed in this study.

These results are consistent with previous studies evaluating vaginal products containing *Coriolus versicolor*. Criscuolo et al. and Serrano et al. reported significant improvements in HPV clearance and cytology normalization after six months of treatment with a *Coriolus versicolor*-based vaginal gel, with clearance rates exceeding those of untreated controls [18,24]. Similarly, Galvez et al. and Serrano et al. observed substantial regression of low-grade cervical lesions following daily application of a similar product, attributing this effect to enhanced epithelial repair and restoration of mucosal immunity [19,25]. In a multicenter randomized prospective trial, Serrano et al. and Cortés et al. found improved clearance of high-risk HPV genotypes and reductions in cervical inflammation after treatment with a multi-ingredient vaginal gel containing *Coriolus versicolor*, hyaluronic acid, and antioxidants [18,19]. The current study corroborates these findings and extends the evidence by demonstrating a robust effect, particularly for HPV 16 and HPV 18, confirmed by tissue-level outcomes.

An additional relevant finding of the present study is the analysis according to HPV infection type. At baseline, the proportions of single-type and multiple-type HPV infections were comparable between the two groups (single-type: 51.0% in the Papilocare group vs. 47.7% in controls; multiple-type: 49.0% vs. 52.3%; *p* = 0.414), supporting baseline comparability with respect to infection multiplicity (Table 1). During follow-up, significantly higher clearance rates were observed in the Papilocare group for both infection patterns. Among women with single-type HPV infection, overall clearance was 83.7% with Papilocare versus 35.0% in controls. Among those with multiple-type HPV infection, overall clearance appeared numerically higher in women with multiple-type infection (95.2% vs. 52.2%), with both comparisons reaching statistical significance (*p* < 0.001) (Table 2).

The pattern observed in multiple-type infections deserves cautious interpretation. In univariate regression analysis, multiple-type HPV positivity was associated with higher odds of clearance (OR 1.75, 95% CI 1.24–2.46; *p* = 0.002). However, this association did not remain statistically significant after adjustment in the multivariable model (aOR 0.50, 95% CI 0.23–1.08; *p* = 0.078) (Table 3). This suggests that infection multiplicity alone may not represent an independent predictor of HPV clearance when treatment use and other clinical variables are taken into account. Therefore, although overall clearance appeared numerically higher in women with multiple-type infections, this finding should not be overinterpreted as evidence that multiple infections are intrinsically associated with a better prognosis. Rather, the results support that the beneficial effect of Papilocare was observed regardless of whether women had single-type or multiple-type HPV infection.

**Figure 2:**
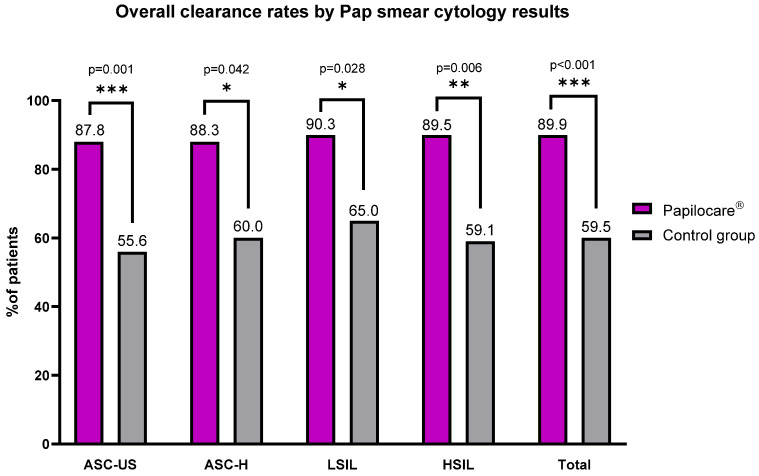
Overall (partial and complete) cytological clearance rates according to baseline cervical cytology in the Papilocare and control groups. *** *p* ≤ 0.001; ** *p ≤* 0.01, * *p* < 0.05 (0.001 < *p* < 0.05).

The cytological results provide further evidence of the effectiveness of the *Coriolus versicolor*-based vaginal gel, as clearance rates for ASC-US, ASC-H, LSIL, and HSIL were markedly higher in the treatment group than in controls (*p* < 0.001) (Figure 2). Notably, overall cytological clearance was consistently higher across all lesion categories in women treated with the gel, with clearance rates of 87.8% for ASC-US, 88.3% for ASC-H, 90.3% for LSIL, and 89.5% for HSIL, compared with 55.6%, 60.0%, 65.0%, and 59.1% in the control group, respectively (Figure 2). These findings suggest that the gel facilitates both viral elimination and accelerated epithelial repair. These findings align with previous studies indicating that the *Coriolus versicolor*-based vaginal gel promotes cytological normalization, enhancing tissue regeneration. Criscuolo et al. [24] observed significant regression of ASC-US and LSIL cytology following treatment, while Bordoy et al. [19] reported notable improvements in cytological abnormalities in a multicenter study of women with low-grade lesions. Bordoy et al. demonstrated that high-risk HPV-positive women treated with a *Coriolus versicolor*-based gel exhibited greater normalization of ASC-US and LSIL cytology than controls, attributing this effect to the balancing of the vaginal microbiota allowing the restoration of cervical microenvironmental balance [19]. These results are consistent with broader evidence that barrier-repairing agents can promote regression of cervical lesions across the Bethesda spectrum. The observed concordance between smear clearance and HPV DNA clearance in the present study further reinforces the therapeutic potential of *Coriolus versicolor*-based vaginal gel in the non-ablative management of HPV-related cytological abnormalities.

Multivariable analysis further reinforced the central role of treatment. Use of the *Coriolus versicolor*–based vaginal gel emerged as the strongest independent factor associated with HPV clearance in the combined cohort, with an adjusted odds ratio of 10.19 (95% CI 3.52–29.47; *p* < 0.001) (Table 3). By contrast, age, HPV vaccination, baseline HPV group, and HPV infection type were not independently associated with clearance after adjustment. Interestingly, multiparity remained independently associated with the outcome in the adjusted model (aOR 0.34, 95% CI 0.13–0.88; *p* = 0.026), whereas other demographic and reproductive characteristics did not show independent effects (Table 3). Overall, these results support the interpretation that the observed differences in clearance were primarily treatment-related rather than being explained by baseline differences in patient characteristics.

While the retrospective design limits causal inference, the large sample size, stringent inclusion criteria, and consistency across HPV DNA, cytology, and tissue-level assessments enhance the credibility of the observed associations. The lack of significant baseline differences in key demographic variables further reduces the likelihood of confounding. Nonetheless, these findings should be interpreted in light of certain limitations. Retrospective data may be subject to incomplete documentation, and the influence of unmeasured behavioral factors, such as sexual practices or condom use, cannot be fully excluded. Additionally, although the six-month follow-up period is clinically relevant, longer-term assessments are necessary to evaluate recurrence and the durability of viral clearance.

In summary, this study demonstrates that *Coriolus versicolor*-based vaginal gel significantly improves clearance of high-risk HPV, particularly HPV 16 and 18, and normalizes cervical cytology. This gel may serve as an effective adjunct to surveillance for women seeking non-ablative treatments. Ongoing randomized trials are required to confirm efficacy, determine optimal use, and assess long-term benefits for HPV management.

The consistency of effects across multiple clinical endpoints reinforces the evidence supporting this non-ablative therapeutic approach. Given its favorable safety profile and ease of use, the *Coriolus versicolor*-based vaginal gel may represent a valuable adjunct in the management of HPV-positive women, particularly for those seeking to avoid immediate excisional procedures or those under surveillance for low-grade lesions.

Future prospective, randomized controlled trials with extended follow-up are necessary to confirm these findings, evaluate long-term viral suppression, and determine the optimal duration of therapy. As the field advances toward individualized, less invasive strategies for HPV management, immunomodulatory bioactive formulations, such as *Coriolus versicolor*-based vaginal gels, may meaningfully contribute to clinical practice and women’s health.

### 4.1. Strengths and Limitations

This study has several notable strengths and limitations. A major strength is the relatively large sample size, which exceeds that of many previous real-world observational studies evaluating non-ablative interventions for HPV infection. The inclusion of 600 women enabled robust comparisons between treatment and control groups and allowed consistent evaluation across multiple HPV genotypes and cytological categories. Moreover, the concurrent assessment of HPV DNA clearance and cytological outcomes provides complementary clinical insight into treatment effects.

Several limitations should be acknowledged. The retrospective design limits causal inference and is inherently susceptible to incomplete documentation and residual confounding. In addition, the limited number of patients in certain subgroups may limit the reliability of subgroup analyses. The inability to perform fully adjusted multivariable analyses further constrains control of potential confounders. Finally, although the six-month follow-up period is clinically meaningful for assessing short-term HPV clearance, it does not allow evaluation of long-term viral persistence or recurrence.

### 4.2. Implications for Practice and Future Research

Despite these limitations, the findings have relevant implications for clinical practice and future research. The consistently higher rates of HPV clearance and cytological normalization observed in the Papilocare group suggest that this *Coriolus versicolor*–based vaginal gel may serve as a useful adjunct to surveillance strategies in women with HPV infection, particularly for those preferring conservative, non-ablative management.

These findings are consistent with prior clinical and real-world evidence supporting the role of vaginal therapies in facilitating HPV clearance and cervical epithelial repair. Given the challenges of spontaneous HPV clearance and the burden of prolonged surveillance, such interventions may help bridge the gap between observation and invasive treatment.

Future studies should prioritize prospective, randomized controlled designs with extended follow-up to confirm the durability of response and assess recurrence. Further stratified analyses by age, HPV genotype, and baseline cytological severity, as well as evaluation of combination approaches with HPV vaccination, would help refine patient selection and optimize clinical management pathways.

## 5. Conclusions

This retrospective cohort study demonstrates that the use of a *Coriolus versicolor*–based vaginal gel is significantly associated with higher rates of HPV clearance and cervical cytological normalization compared with standard clinical follow-up alone. The consistency of these findings across virological and cytological endpoints, together with multivariable analyses identifying Papilocare use as the strongest independent predictor of HPV clearance, supports the potential clinical value of this non-ablative, locally applied intervention.

Although the retrospective design and limited follow-up duration preclude definitive causal conclusions, the large sample size and comparable baseline characteristics between groups strengthen the reliability of the observed associations. Overall, the *Coriolus versicolor*–based vaginal gel may represent a safe and effective adjunct to current HPV management strategies, bridging the gap between observation and invasive approaches.

Prospective, randomized controlled trials with longer follow-up are warranted to confirm these results and further define its role within cervical cancer prevention and surveillance algorithms.

## Figures and Tables

**Table 1:** Distribution of age, HPV genotype positivity and Pap smear cytology between study groups.

	Papilocare (n = 300)	Control (n = 300)	*p*-Value
**Age (years), mean ± SD**			0.279 ^m^
	37.2 ± 9.5	38.0 ± 9.5	
**Age group (years)**			0.612 ^c^
<30	70 (23.3%)	64 (21.3%)	
30–45	173 (57.7%)	169 (56.3%)	
>45	57 (19.0%)	67 (22.4%)	
**HPV Genotype Distribution**			***p*-value**
			0.124 ^c^
HPV 16	113 (37.7%)	118 (39.3%)	
HPV 18	23 (7.7%)	27 (9.0%)	
Other High-risk HPV Types	112 (37.3%)	100 (33.3%)	
Intermediate-risk HPV Types	17 (5.7%)	31 (10.3%)	
Low-risk HPV Types	35 (11.6%)	24 (8.0%)	
**HPV Infection Type Distribution**			***p*-value**
			0.414 ^c^
Single-type HPV	153 (51.0%)	143 (47.7%)	
Multiple-type HPV	147 (49.0%)	157 (52.3%)	
	**Papilocare (n = 260)**	**Control (n = 271)**	***p*-value**
**Cytology**			0.171 ^c^
Normal Cytology	125 (48.1%)	140 (51.7%)	
ASC-US	49 (18.8%)	54 (19.9%)	
ASC-H	17 (6.5%)	15 (5.5%)	
LSIL	31 (11.9%)	40 (14.8%)	
HSIL	38 (14.6%)	22 (8.1%)	

Data are presented as mean ± standard deviation or n (%), as appropriate. Percentages are reported as column percentages. Each patient was classified according to the predominant HPV genotype. Continuous variables (age) were compared using the Mann–Whitney U test. Categorical variables (age group, HPV genotype distribution, HPV infection type, and cytological findings) were compared using the chi-square test or Fisher’s exact test, as appropriate based on expected cell counts. ^m^ Mann–Whitney U test; ^c^ chi-square test.

**Table 2:** HPV and Cytology Clearance According to Study Groups (Complete/Partial/Overall).

	CLEARANCE								
	Papilocare (n = 300)				Control (n = 300)				
	Complete	Partial	Overall	No	Complete	Partial	Overall	No	*p*-value
**HPV Genotype**									
HPV 16	87 (77.0%)	16 (14.2%)	103 (91.2%)	10 (8.8%)	30 (25.4%)	19 (16.1%)	49 (41.5%)	69 (58.5%)	<0.001 ^c^
HPV 18	17 (73.9%)	4 (17.4%)	21 (91.3%)	2 (8.7%)	5 (18.5%)	7 (25.9%)	12 (44.4%)	15 (55.6%)	0.001 ^f^
Other High-risk HPV Types	86 (76.8%)	14 (12.5%)	100 (89.3%)	12 (10.7%)	25 (25.0%)	21 (21.0%)	46 (46.0%)	54 (54.0%)	<0.001 ^c^
Intermediate-risk HPV Types	13 (76.5%)	2 (11.8%)	15 (88.2%)	2 (11.8%)	6 (25.0%)	4 (16.7%)	10 (41.7%)	14 (58.3%)	0.012 ^f^
Low-risk HPV types	22 (62.9%)	7 (20.0%)	29 (82.9%)	6 (17.1%)	7 (22.6%)	8 (25.8%)	15 (48.4%)	16 (51.6%)	0.018 ^f^
Total	225 (75.0%)	43 (14.3%)	268 (89.3%)	32 (10.7%)	73 (24.3%)	59 (19.7%)	132 (44.0%)	168 (56.0%)	<0.001 ^c^
**HPV Infection Type**									
Single-type HPV	128 (83.7%)	0 (0.0%)	128 (83.7%)	25 (16.3%)	50 (35.0%)	0 (0.0%)	50 (35.0%)	93 (65.0%)	<0.001 ^c^
Multiple-type HPV	97 (66.0%)	43 (29.3%)	140 (95.2%)	7 (4.8%)	23 (14.6%)	59 (37.6%)	82 (52.2%)	75 (47.8%)	<0.001 ^c^
**Pap Smear Cytology**									
	**Papilocare (n = 135)**				**Control (n = 131)**				
	**Complete**	**Partial**	**Overall**	**No**	**Complete**	**Partial**	**Overall**	**No**	***p*-value**
ASC-US	43 (87.8%)	0 (0%)	43 (87.8%)	6 (12.2%)	30 (55.6%)	0 (0%)	30 (55.6%)	24 (44.4%)	0.001 ^f^
ASC-H	13 (76.5%)	2 (11.8%)	15 (88.3%)	2 (11.7%)	4 (26.7%)	5 (33.3%)	9 (60.0%)	6 (40.0%)	0.042 ^f^
LSIL	21 (67.7%)	7 (22.6%)	28 (90.3%)	3 (9.7%)	18 (45.0%)	8 (20.0%)	26 (65.0%)	14 (35.0%)	0.028 ^c^
HSIL	25 (65.8%)	9 (23.7%)	34 (89.5%)	4 (10.5%)	6 (27.3%)	7 (31.8%)	13 (59.1%)	9 (40.9%)	0.006 ^f^
Total	102 (75.6%)	18 (13.3%)	120 (89.9%)	15 (11.1%)	58 (44.3%)	20 (15.3%)	78 (59.5%)	53 (40.5%)	<0.001 ^c^

Data are presented as n (%), with percentages calculated within each study group. Each patient was classified according to the predominant HPV genotype. Row-specific *p*-values represent comparisons of clearance status (complete, partial, and no clearance) between the Papilocare and control groups within each HPV genotype, infection type, and cytological category. Statistical analyses were performed using the chi-square test (^c^) or Fisher’s exact test (^f^), as appropriate based on expected cell counts. Adjusted associations are presented in the regression analysis table.

**Table 3:** Factors Associated with HPV Clearance in the Combined Control and Papilocare Cohort (Binary Logistic Regression Analysis).

	Clearance (+), n (%)	Clearance (–), n (%)			Combined Cohort	
Variable			Univariate OR (95% CI)	*p*-Value	Multivariate aOR (95% CI)	*p*-Value
**Papilocare Use**						
No	134 (44.7%)	166 (55.3%)	Reference			
Yes	268 (89.3%)	32 (10.7%)	10.38 (6.74–15.96)	**<0.001**	10.19 (3.52–29.47)	**<0.001**
**Age (years)**						
<30	98 (73.1%)	36 (26.9%)	Reference			
30–45	223 (65.2%)	119 (34.8%)	1.445 (0.849–2.459)	0.175		
>45	81 (65.3%)	43 (34.7%)	0.995 (0.646–1.532)	0.981	0.75 (0.35–1.60)	0.452
**Pregnancy**						
Nulliparous	130 (87.2%)	19 (12.8%)	Reference			
Multiparous	164 (73.5%)	59 (26.5%)	2.461 (1.398–4.335)	**0.002**	0.34 (0.13–0.88)	0.026
**Menstrual Status**						
Regular	196 (77.2%)	58 (22.8%)	Reference			
Irregular	70 (88.6%)	9 (11.4%)	1.560 (0.568–4.285)	0.389		
Menopause	13 (68.4%)	6 (31.6%)	3.590 (1.091–11.806)	**0.035**	1.26 (0.65–2.44)	0.490
**Age at menarche (years)**						
≤15	225 (80.6%)	54 (19.4%)	Reference			
>15	14 (73.7%)	5 (26.3%)	1.49 (0.51–4.31)	0.464	0.58 (0.11–3.17)	0.533
**First Intercourse (years)**						
≤18	60 (88.2%)	8 (11.8%)	Reference			
>18	209 (78.9%)	56 (21.1%)	2.010 (0.91–4.45)	0.085	0.47 (0.17–1.26)	0.132
**HPV Vaccination**						
No	114 (44.9%)	140 (55.1%)	Reference			
Yes	275 (83.6%)	54 (16.4%)	0.160 (0.109–0.234)	**<0.001**	1.23 (0.42–3.63)	0.704
**HPV Positivity by Infection Type**						
Single-type HPV	178 (60.1%)	118 (39.9%)	Reference			
Multiple-type HPV	224 (73.7%)	80 (26.3%)	1.75 (1.24–2.46)	**0.002**	0.50 (0.23–1.08)	0.078
**Baseline HPV Group**						
Others	333 (67.5%)	160 (32.5%)	Reference			
HPV 16, 18 and Other High-Risk Types	69 (64.5%)	38 (35.5%)	1.146 (0.739–1.777)	0.542	0.58 (0.24–1.45)	0.247

*p*-values were derived from univariate and multivariable binary logistic regression analyses using the Wald test. The outcome variable was HPV clearance (clearance [+] vs. clearance [−]). Values are presented as n (%). OR: odds ratio; aOR: adjusted odds ratio; CI: confidence interval.

## Data Availability

The data presented in this study are not publicly available due to ethical and privacy restrictions related to patient confidentiality. Anonymized data supporting the findings of this study are available from the corresponding author upon reasonable request and with permission from the relevant institutional ethics committee.

## Abbreviations

The following abbreviations are used in this manuscript:

HPV	Human Papillomavirus
LEEP	Loop electrosurgical excision procedure
ASC-US	Atypical squamous cells of undetermined significance
ASC-H	Atypical squamous cells—cannot exclude high-grade squamous intraepithelial lesion
LSIL	Low-grade squamous intraepithelial lesions
HSIL	High-grade squamous intraepithelial lesions
CIN	Cervical intraepithelial neoplasia
